# Multiwalled carbon nanotube hybrids as MRI contrast agents

**DOI:** 10.3762/bjnano.7.102

**Published:** 2016-07-27

**Authors:** Nikodem Kuźnik, Mateusz Michał Tomczyk

**Affiliations:** 1 Silesian University of Technology, Faculty of Chemistry, M. Strzody 9, 44-100 Gliwice, Poland

**Keywords:** carbon nanotube hybrids, contrast agent, molecular probe, multiwalled carbon nanotubes (MWCNT), magnetic resonance imaging (MRI), relaxation

## Abstract

Magnetic resonance imaging (MRI) is one of the most commonly used tomography techniques in medical diagnosis due to the non-invasive character, the high spatial resolution and the possibility of soft tissue imaging. Contrast agents, such as gadolinium complexes and superparamagnetic iron oxides, are administered to spotlight certain organs and their pathologies. Many new models have been proposed that reduce side effects and required doses of these already clinically approved contrast agents. These new candidates often possess additional functionalities, e.g., the possibility of bioactivation upon action of particular stimuli, thus serving as smart molecular probes, or the coupling with therapeutic agents and therefore combining both a diagnostic and therapeutic role. Nanomaterials have been found to be an excellent scaffold for contrast agents, among which carbon nanotubes offer vast possibilities. The morphology of multiwalled carbon nanotubes (MWCNTs), their magnetic and electronic properties, the possibility of different functionalization and the potential to penetrate cell membranes result in a unique and very attractive candidate for a new MRI contrast agent. In this review we describe the different issues connected with MWCNT hybrids designed for MRI contrast agents, i.e., their synthesis and magnetic and dispersion properties, as well as both in vitro and in vivo behavior, which is important for diagnostic purposes. An introduction to MRI contrast agent theory is elaborated here in order to point to the specific expectations regarding nanomaterials. Finally, we propose a promising, general model of MWCNTs as MRI contrast agent candidates based on the studies presented here and supported by appropriate theories.

## Introduction

The interdisciplinary character of nanotechnology crossing over domains of chemistry, physics, material science, engineering, biology and medicine has been somewhat harnessed to enhance both the quality and safety of human life. Parallel investigations at both a macroscopic and nano-/molecular level have already led, or at least are close to, myriads of applications. Medicine is one such field in which nanomaterials can help in therapy and diagnosis. Yet the unusual features of nanomaterials have raised natural concerns, especially regarding potential medical applications because of their exogenous origin and unknown physiology. Nevertheless, the unique combination of features that is found in nanoparticles has opened up a new era of biomaterials. These offer several advantages of great importance to biomedical applications, e.g., the possibility of appropriate size and shape designed for the desired target, modulation of their biocompatibility and thus biodistribution which is followed, finally, by surface modifications leading to amphiphilic or specifically targeted behavior. Moreover, their magnetic, electric and photoluminescence properties can be adjusted and exploited for desired modalities. There are visions on the application of carbon nanotubes (CNT) drug and genetic material delivery, immunotherapy or photothermal cancer therapy [1–2]. The 'quantum leap' [3] of bionanomaterials has also affected magnetic resonance imaging (MRI). This technique, which has already matured into a basic diagnostic tool in medicine, has an edge over other tomographic methods due to its non-invasive character, the high spatial and temporal resolution and the possibility of analyzing soft tissues. The substitution of X-rays (as used in computer tomography) by a magnetic field that causes no side effects and has no limit to penetration depth into the body (unlike optical methods) together with information on the physicochemical state of the organs and the ability to follow the motion of fluids (magnetic resonance angiography) make the technique even more universal. Moreover, MRI has found its applications in manufacturing and in the food industry. Medical diagnosis additionally benefits from contrast agents (CAs) in the MRI technique via additionally administered formulations, thus leading to enhanced sensitivity by highlighting particular objects in the images; the potential identification of pathologies may result in earlier tumor diagnosis. Despite the currently used gadolinium MRI CAs, administered to ca. 10 million patients each year worldwide [4], investigations have focused on this lanthanide metal in order to reduce the side effects and needed dose, but also to enhance the selectivity of desired organs and tissues as well as to allow for additional activity in other techniques (e.g., computed tomography (CT), positron emission tomography PET and optical methods). It has been shown that nanomaterials could be more effective than classical CAs by 2–100 times in their activity and lead to dose reductions by 1–2 orders of magnitude. Moreover, the abovementioned features of nanoparticles and the possibility to penetrate cell membranes as well as coupling with drugs create a very attractive vision of future applications not only in diagnosis but also to monitor physiology and therapeutic progress. There have been numerous investigations on applications of carbon nanomaterials in bioimaging [4–9], e.g., graphene, graphite oxide with manganese residues [10], gadolinium anchored on fullerenes [11], and nanodiamonds [12]. Sitharaman’s and Wilson’s discoveries of gadonanotubes, such as ultrashort single-wall carbon nanotubes (US-SWCNTs) enriched with Gd^3+^, have established a new and promising model of nanostructural MRI CAs, which that is very efficient in the *T*_1_ mode [3,13–14]. Then single-walled carbon nanotubes (SWCNTs) were functionalized in various ways and investigated as MRI CAs [15]. Gadolinium was introduced in the form of diethylene triamine pentaacetic acid (DTPA) complexes (classical CAs) on SWCNTs [16], superparamagnetic iron oxides (SPIOs) were anchored on SWCNTs [6] and, on the contrary, iron-deficient SWCNTs [17] were found to exhibit good properties for potential MRI CAs. These first works in the realm of CNTs showed great potential but also the problems and imperfections that needed to be overcome. One of the first challenges was to reduce the tendency of CNTs for aggregation, which seriously affected the stability of their aqueous and buffered dispersions. Another issue was to enhance their potential as CAs exclusively in one of the MRI modes (*T*_1_ or *T*_2_). Further requirements consisted in better biocompatibility with the targeting of tumor cells, coupling with stem cells as well as crossing the cell membrane and blood–brain barrier. Finally, involving CNT activity in other diagnostic techniques (fluorescence imaging, PET and others) is an attractive approach to administering one formulation for different tomography and therapeutic purposes. These issues, achieved by (non-)covalent modifications of pristine multiwalled carbon nanotubes (MWCNTs) and oxidized nanotubes are described in this review. We present an introduction to the MRI technique and to the mechanism of CA action followed by synthetic routes of MWCNT pretreatment with oxidizing agents and layer-by-layer decoration (LBL) with (non-)ionic electrolytes. Functionalization of super/paramagnetic nanoparticles and the resulting properties are described. Finally, toxicity and in vivo MRI effects are discussed. Some researchers have already pointed to poor consistency of the data presented in the literature which results from missing information regarding both the composition and morphology of CNTs in all stages of their transformations [18]. Surprising results of the relaxation effects both in vitro and in vivo and depending on a number of parameters, such as content of the residual catalyst, size of the CNTs or "wrapping media" (the electrolyte used to stabilize the dispersions), were also reported [18–19]. We discuss these problems here and support them with the available relaxation theories and the associating factors which may be helpful in designing new and even better models of MWCNT MRI CAs.

## Review

### 1 Specificity of MWCNT hybrids for the use as MRI contrast agents

#### Synthesis

**Figure 1:**
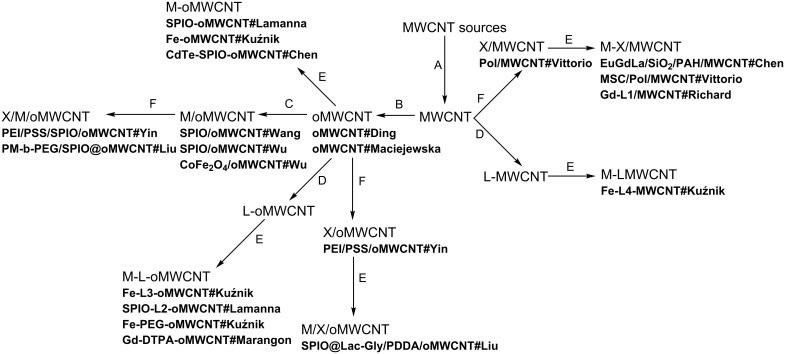
Transformations of MWCNTs. Only the final products subjected to relaxometric or MRI studies are presented in the figure with appropriate abbreviations. A - synthesis of MWCNTs with different catalysts, B - oxidation of MWCNTs with HNO_3_ (and in some cases with H_2_SO_4_), C - non-covalent SPIO introduction, D - covalent (and coordination) bonding of organic ligands, E - introduction (by coordination or other interactions) of metal species: ions, oxides, stem cells, F - non-covalent wrapping (X stands for a wrapping medium).

**Figure 2:**
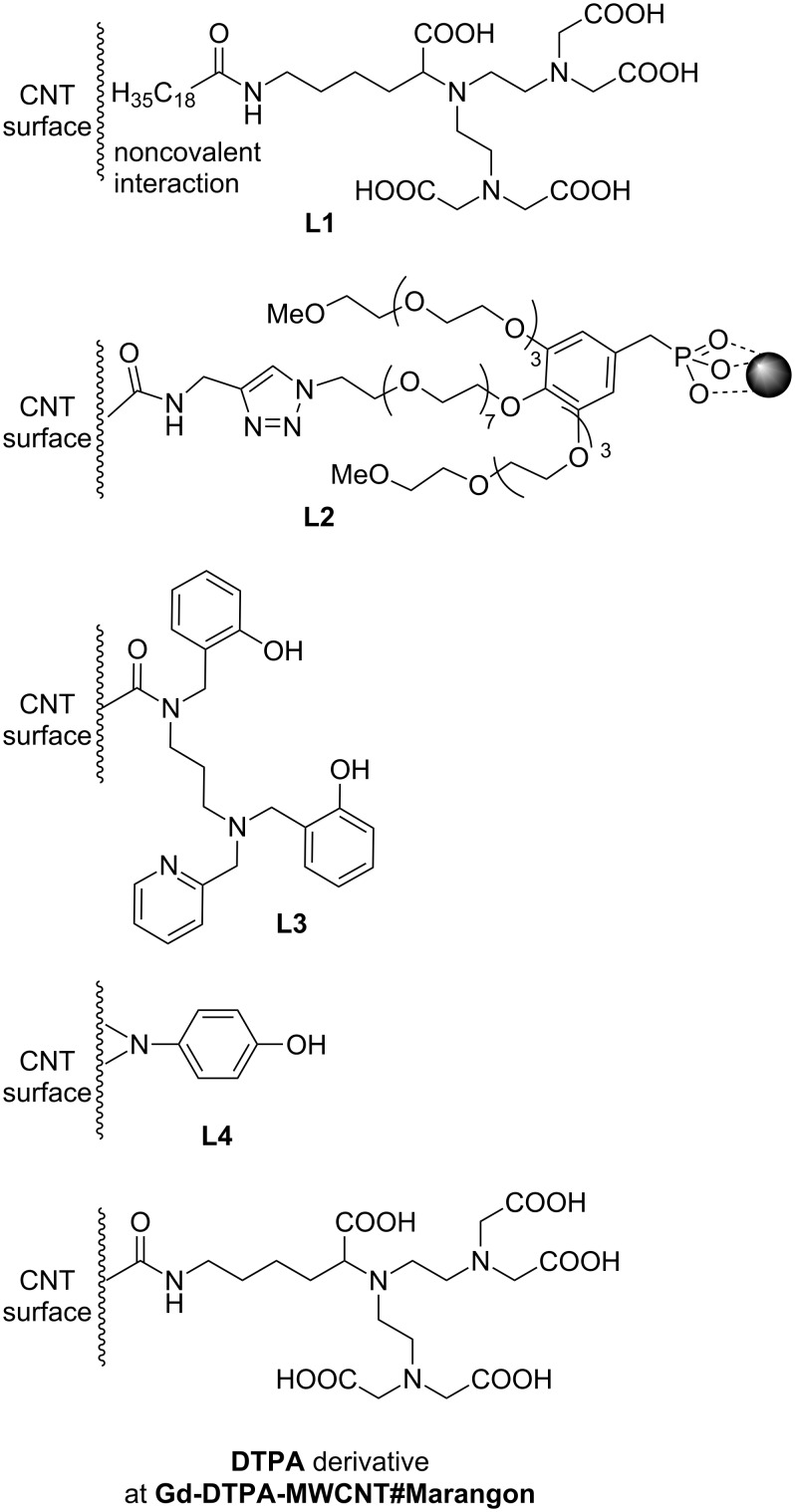
Organic ligands in MWCNT hybrids.

**Figure 3:**
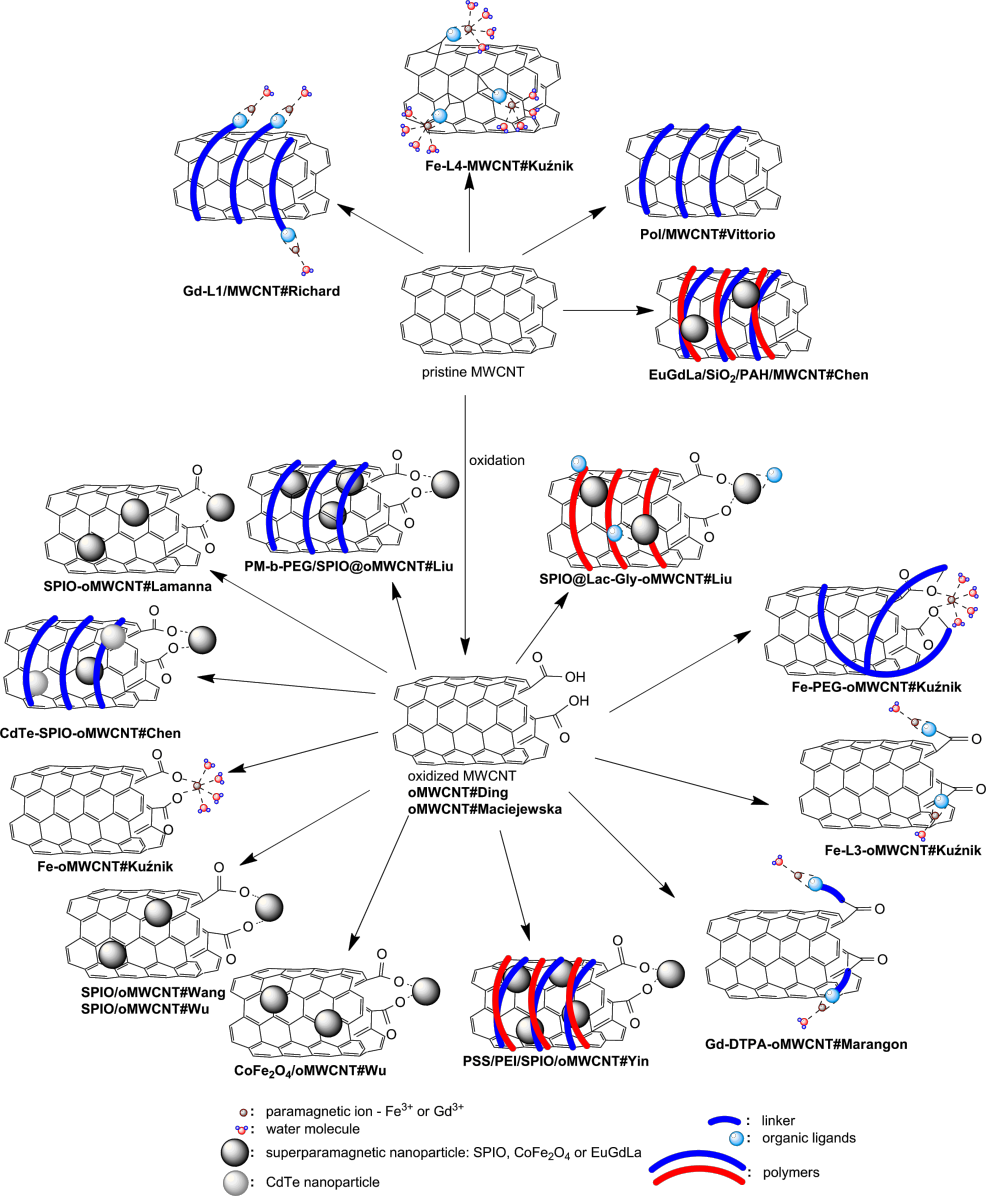
Structure of MWCNT hybrids.

MWCNTs were synthesized by chemical catalytic vapor deposition (c-CVD) [20]. This is the most common method of MWCNT synthesis. The inherent consequence of the application of a metal catalyst (e.g., ferrocene, aluminum oxide) is the presence of a nanometallic deposit in the tubes [21]. Thus obtained MWCNTs are already an interesting scaffold for both (non-)covalent modifications leading to CA candidates (Figure 1, Figure 2 and Figure 3).

One of the first reports of the application of a MWCNT as a CA, published by Richard in 2008, describes the non-covalent decoration of MWCNT with a classical gadolinium ligand – the DTPA derivative [16]. The ligand was coupled with a lipid chain, which was expected to enhance adsorption on the surface of the nanotube. The heptadentate DTPA ligand (**L1**), in turn, secured permanent coordination of Gd^3+^ in the new hybrid **Gd-L1/MWCNT#Richard**. Vittorio non-covalently combined pristine MWCNT with mesenchymal stem cells (MSCs) [22]. MSCs labeled with MWCNT hybrids (**MSC/Pol/MWCNT#Vittorio**) were obtained by mixing Pluronic^®^ F127 dispersion of MWCNTs (**Pol/MWCNT#Vittorio**) with incubated MSCs. One-month stability of the resulting dispersion was proven and a permanent binding of MWCNTs to the cells was observed in in vivo studies. It is not clear whether the polyether chains of the poloxamer Pluronic^®^ served as a non-ionic wrapping agent securing solid anchoring of the MWCNTs on the cell membrane [23–24]. Alternatively, its role might be more focused on stabilizing a disperse system by preventing the CNTs from agglomeration, while the lipophilic surface of the MWCNT has sufficiently high affinity to the cell membrane for permanent connections. Chen subjected pristine MWCNT to LBL (layer-by-layer) non-covalent functionalizations with the polyelectrolyte poly(allylamine hydrochloride) (PAH) followed by silica coating with europium, gadolinium and lanthanum fluoride co-precipitation (**EuGdLa/SiO_2_/PAH/MWCNT#Chen**) [25]. The authors claimed that fluoride nanocrystals (5–10 nm) coated the MWCNT effectively due to high electrostatic attraction from the polyelectrolyte. The silica layer was introduced to prevent photoluminescence quenching. This functionalization allows for europium photoluminescence emission and endows the hybrids with a second, optical modality. We took another approach to MWCNT functionalization by introducing *N*-phenylaziridine groups on the carbon scaffold [26] in the reaction of aryl azide with pristine MWCNT [27]. The reaction of *p*-azidophenol with MWCNT dispersion resulted in a hybrid containing 9% (w/w) of N–Ph–OH according to TGA. Since phenols exhibit a high affinity and selectivity for Fe^3+^ [28], we coordinated the iron ions to achieve an Fe content over 6% (m/m) in **Fe-L4-MWCNT#Kuźnik**. Despite the huge possibilities of other MWCNT functionalizations [29–30], the majority of transformations begin in the fragmentation and rapture of the aromatic skeleton with oxidizing acids [31]. In a majority of works, the typical nitrating mixture of mixed acids HNO_3_ and H_2_SO_4_ is applied in the classical ratio of 1:3 (v/v) under reflux for 4–24 h [18,26,32–33], or at room temperature, although under sonication for 16–24 h [34–37]. Alternatively, reflux in sole 15–20% HNO_3_ for 6–45 h is performed [38–40]. This approach introduces oxygen, polar and, in most cases, protic acidic groups and opens up many possibilities for further functionalization, decoration, substitution or direct coordination of metals and their compounds. Carboxylic, hydroxyl (both phenol and alkyl alcohol) and carbonyl groups are formed on the surface and edges of the oxidized nanotubes **oMWCNT**. These materials, **oMWCNT#Maciejewska**, were investigated by Maciejewska [18]. A different catalyst (ferrocene) percentage (2 wt %, 5 wt %, 10 wt %) in the c-CVD procedure resulted in corresponding residual iron contents of 3.9, 5.8 and 12.4 wt %, as well as in varying diameters of 29, 49 and 40 nm, respectively. Similar studies were presented by Ding (**oMWCNT#Ding**) [35]. Functional groups on the oMWCNT surface exhibit Lewis-base character and therefore already have a sufficient affinity to metals and their oxides to serve as coordinating sites. It is interesting to report Liu’s further development of COOH groups by reaction of **oMWCNT** with citric acid (2 h at 80 °C) [33]. The presence of several oxygen groups in the neighborhood, which is a common situation for the disrupted aromatic skeleton, additionally supported by the presence of π-electrons forms a chelating cavity for the metal ion. Lamanna used such a model for coordination of the iron oxide nanoparticles (**SPIO-oMWCNT#Lamanna**) [37]. This model was obtained by ligand exchange of SPIO–fatty acid salts to the acidic surface of the **oMWCNT**. Covalent bonding, presented by Chen, was also done by dehydrating crosslinking [40]. First, SPIO was bonded to the PAH-covered **oMWCNT**, followed by the introduction of CdTe quantum dots leading to **CdTe-SPIO-oMWCNT#Chen** nanohybrids. Finally, we showed that oxidized nanotubes are attractive for hard Lewis acids, e.g., Fe^3+^ ions, and form stable nanomolecular complexes, namely **Fe-oMWCNT#Kuźnik** [41]. Further covalent functionalization of **oMWCNT** is aimed at introducing specific organic ligands to permanently chelate metal ions or their oxides. This is the manner in which Lamanna prepared an oligoglycol dendron with phosphonic groups responsible for SPIO anchoring. The ligand was introduced on the alkyne-derived **oMWCNT** by a click reaction in **SPIO-L2-oMWCNT#Lamanna** [37]. A classical DTPA ligand was used to functionalize **oMWCNT** by Marangon in an amidation reaction with a protected DTPA derivative equipped with an NH_2_-spacer [36]. The product was then used to anchor Gd^3+^ ions, thus a CNT-supported version of a common gadolinium contrast agent was obtained (**Gd-DTPA-oMWCNT#Marangon**). We utilized this approach to build a CNT-derivative of Fe^3+^ complexes with amino-phenol ligands, which showed promising results as *T*_1_ contrast agents [42]. An in situ generated acid chloride of **oMWCNT** was reacted with the ligand, resulting in an Fe^3+^ sponge, **Fe-L3-oMWCNT#Kuźnik**, able to absorb 14% iron over its entire mass [26]. We used the same acid chloride of the **oMWCNT** intermediate to covalently bond PEG–OH (PEG: poly(ethylene glycol)) chains and then to anchor the iron cations (**Fe-PEG-oMWCNT#Kuźnik**). Finally, the last group of **oMWCNT** modifications consisted in non-covalent functionalization. Direct in situ generation of superpara- and ferromagnetic species in the presence of these derivatives of **oMWCNT** were reported. Wu co-precipitated Fe(II) and Fe(III) chlorides with NaOH in oMWCNT dispersion, obtaining a nanocrystalline deposit of Fe_3_O_4_ (**SPIO/oMWCNT#Wu**) [38], while Wang used the thermal annealing method of iron(II) acetate (**SPIO/oMWCNT#Wang**) [43]. Solvothermal co-precipitation of CoCl_2_ and FeCl_3_, also by Wu, led to the non-covalent deposition of magnetic cobalt ferrite (**CoFe_2_O_4_/oMWCNT#Wu**) [39]. It was found that a low temperature (180 °C) allowed for the production of hybrids uniformly coated with metallic superparamagnets, yet an increased temperature was responsible for agglomeration of the nanoparticles on the nanotube walls. Yin, finally, presented a method of SPIO deposition by thermal decomposition of ferrocene under aerobic conditions [34]. The so-obtained hybrids were further wrapped with anionic polyelectrolyte polystyrene sulfonate (PSS) and then with cationic polyethylenimine (PEI). A modification of this hybrid, **PEI/PSS/SPIO/oMWCNT#Yin**, was additionally synthesized by covalent carboxylic-amine crosslinking with folic acid (FA) (**FA-PEI/PSS/SPIO/oMWCNT#Yin**). A reversed methodology was applied for the formation of two other hybrids, where the initially wrapped **oMWCNT** with polyelectrolytes was used to support magnetic nanoparticles. Chen published a magnetic-fluorescent model, **EuGdLa/SiO_2_/PAH/MWCNT#Chen**, which resulted from the in situ precipitation of SiO_2_ on PAH non-covalently coated **oMWCNT** followed by co-precipitation of lanthanide fluorides [25]. In Liu’s work, the sonication product of oMWCNT with poly(diallyldimethylammonium chloride) (PDDA) was used to anchor SPIO@Lac-Gly yielding **SPIO@Lac-Gly/PDDA/oMWCNT#Liu** [32]. Lactose (Lac) was coupled with glycine (Gly) in the Maillard reaction. This biocompatible scaffold was used for co-precipitation of SPIO from iron(II) and iron(III) chlorides. Liu recently presented the possibility of incorporating SPIO into the oMWCNT [33]. This was achieved by the following sequence: sonication of oMWCNT with Fe(acac)_3_ in ethanol, solvent evaporation, partial reduction of Fe(acac)_3_ to Fe_3_O_4_ with hydrazine by ultrasonication for 10 min and irradiation by microwave at 100 °C for 20 min. **PM-b-PEG/SPIO@oMWCNT#Liu** hybrids were obtained by non-covalent decoration with the amphiphilic, bipolar diblock copolymer poly[2-(methacryloyloxy)ethyltrimethylammonium chloride]-*block*-poly(ethylene glycol) monomethacrylate (PMETAC-*b*-PEGMA).

#### Characterization of MWCNT hybrids

The hybrids were investigated using several methods. The morphology is well visible in transmission electron microscopy (TEM) images. In a majority of the cases, complete and homogeneous coating with nanoparticles of 5–10 nm in size was revealed in the images [25,32,34,39,43]. After loading SPIO on the oMWCNT, the nanoparticles were well visible on the nanotubes and the following functionalization with polyelectrolytes could also be observed in the TEM images [33]. High resolution TEM (HRTEM) and X-ray diffraction (XRD) revealed the crystalline structure of the nanoparticles and enabled qualitative identification of the elements (e.g., lanthanides and silicon in **EuGdLa/SiO_2_/PAH/MWCNT#Chen**) and their structural form (i.e., γ-Fe_2_O_3_ and Fe_3_O_4_ in the hybrids with SPIO). Fourier transform infrared spectroscopy (FTIR) and thermogravimetric analysis (TGA), supported again by TEM, were applied to monitor the effects of wrapping with organic moieties. Infrared spectroscopy has commonly been used to follow the covalent transformations, e.g., the data were shown to couple oxidized SWCNT with hyaluronic acid containing amino groups [44]. Liu described interesting mass growth during the heating of SPIO–MWCNT hybrids above 400 °C in TGA, which was explained as oxidation of iron(II) in Fe_3_O_4_ to iron(III) in Fe_2_O_3_, also observed as a characteristic color change from dark to red [32]. We have successfully applied UV–vis analysis to monitor the transformations of MWCNT [26]. For instance, the characteristic band at 232 nm in **oMWCNT** was enriched with a band at 274 nm in **Fe-L3-oMWCNT#Kuźnik** that is specific for organic functional groups of the L3 ligand. In order to assess the charge of species, zeta potential measurements were performed and turned out to be a very informative tool for covering the nanotubes with polyelectrolytes, e.g., a step-by-step analysis gave a slightly negative zeta potential SPIO/oMWCNT, which was lowered to −73.5 mV by covering with the negatively charged PSS and then turned positive to +45.0 mV after the following layer of cationic PEI, resulting in **PEI/PSS/SPIO/oMWCNT#Yin** [34]. However, consequent studies on morphology changes of MWCNT during all of the procedures applied to the materials are rare. Only Marangon stated that the morphology of the nanotube skeleton was not affected by the functionalization [36]. Additionally, Yin, based on XRD and X-ray photoelectron spectroscopy (XPS) measurements, concluded that, once anchored, SPIO nanoparticles are tightly bonded to the nanotube scaffold despite agitation, prolonged stirring or sonication [32]. Thus an assumption has to be made on the stability of the morphology of oMWCNT affected initially only intentionally by pretreatment with oxidizing acids.

The specific steps of order of pretreatment and conditions are different for the various oMWCNT models described here. These various introductory modifications make the relations of synthesis and properties difficult to analyze. The situation is complicated by additional issues, e.g., one specific remark was made in Chen’s work regarding pretreatment of MWCNT [40]. The authors first refluxed pristine MWCNT in 17% HNO_3_ for 38 h and then subjected it to sonication with the mixed acid at 35–40 °C for 4 h in order to, as they stated, cut the nanotubes into short pieces. Finally, the oMWCNTs were treated with a 4:1 mixture of conc. H_2_SO_4_ and 30% H_2_O_2_ at 70 °C for 1 h for “further polishing”. According to Liu’s original report on SWCNT oxidation, the presence of H_2_O_2_ doubles the rate of nanotube shortening [45]. This is one of the examples where the carbon scaffold has been specifically modified and it is difficult to compare it with other, more common pretreatment methodologies, especially regarding the resulting properties that are crucial for applications as MRI CAs.

Another issue is SPIO preparation. Liu and Wu introduced it via co-precipitation of Fe^3+^ and Fe^2+^ ions on MWCNT [32,38]. According to Wang’s work, iron(II) acetate is mixed with oMWCNT dispersion, subjected to thermal decomposition at 500 °C, initially in Ar/H_2_ and in the presence of air as an oxidant [43]. Yin also exploited the oxidative conditions of air by thermal decomposition of a solid MWCNT–ferrocene mixture [34], whereas Lamanna produced coated iron oxides by thermal decomposition of iron(II) stearate in the presence of oleic acid [37]. All of these approaches led to the formation of iron oxides with superparamagnetic properties, as determined by superconducting quantum interference device (SQUID) measurements. Only in a few cases was the real composition proven by X-ray techniques. Nevertheless, these nanoparticles belong to the group of SPIOs known as MRI CAs [46–47], while the resulting behavior after coupling with MWCNT is determined as an entire, new SPIO–MWCNT hybrid. More thorough studies of SPIO impact on the properties and biocompatibility of hybrids should be encouraged.

#### Magnetic properties

The magnetic character of MWCNTs originates from residual metal nanoparticles incorporated during the synthesis. It is further altered by intentional introduction of other magnetic species such as SPIO. The magnetic properties are determined by the superconducting quantum interference (SQUID) technique. Magnetic hysteresis loops close to zero magnetization in a varied magnetic field is typical behavior proving the super- [34,43] or paramagnetic [22] character of the introduced nanoparticles. Initially, the separately produced SPIOs, such as SPIO@Lac-Gly, exhibit higher magnetization, but their anchoring on a nanotube results in a decrease of this value [32]. On the contrary, higher magnetization was recorded for the **CdTe-SPIO-oMWCNT#Chen** hybrid than for the SPIO solely [40]. No remnant magnetization was observed for the samples. Additionally, ZFC/FC curves as temperature functions provide other typical behavior of a superparamagnetic body. Marangon observed a ferromagnetic component for the FC and ZFC magnetization curves which was assigned to the residual nanoparticles left after the catalyst had not sufficiently washed out in the pretreatment procedure [36]. On the other hand, a trivial test for the superparamagnetic properties of nanotube hybrids could be performed with a solid magnet which would attract the material when spread in a powdered form [32,34,38]. This visual effect encourages the exploitation of magnetic properties in synthesis and in therapy. Although they have not yet been applied in MWCNT CA candidates, an example of SWCNT separation in the magnetic field was described by Choi [6]. Nanotubes with SPIO anchored at one of the ends were combined with DNA chains. Then the dispersion of hybrids was placed over a 0.5 T magnetic array for two days. The hybrids arranged according to their magnetic moment (and the content of iron), so the researchers were able to separate mechanically the most iron-enriched hybrids from the rest of the product. Another example comes from Lamanna's research, where enhanced uptake of CNTs by PC3 tumor cells directly above a neodymium magnet compared to a region without an additional magnetic field [37].

#### Behavior of dispersion

The stability of dispersion is the key parameter for the possible application of MWCNT hybrids in medicine. It has even been suggested that non-covalently functionalized gadonanotubes have low dispersibility and for this reason in vivo studies on them are very limited [36]. The **Gd-DTPA-oMWCNT#Marangon** hybrid could reach a concentration of 5 mg/mL in physiological saline and, according to the study, this dispersibility is during circulation with the blood. Stability is usually assessed directly by visual evaluation of the samples in several, especially biocompatible, media, such as water, saline, buffers, plasma, serum, or even cell cultures [18,33–34]. Stable dispersion is obtained by at least several minutes of sonication. Wu proved the stability of **SPIO/oMWCNT#Wu** in water for two weeks [38]. Models with stem cells, e.g., **MSC/Pol/MWCNT#Vittorio** (and other preparations in cell–nanotube studies), were suspended in 1% agarose [22,37]. Since rapid sedimentation is easy to observe, unstable models could instantaneously be eliminated. Some works contained comments on stability for longer periods, e.g., Liu examined the dispersion stability of **PM-b-PEG/SPIO@oMWCNT#Liu** in phosphate-buffered saline (PSB) and in water, thus proving that the presence of the amphiphilic polymer PMETAC-*b*-PEGMA prevents sedimentation [33]. **CoFe_2_O_4_/oMWCNT#Wu** formed a stable aqueous dispersion for 2 weeks [39]. Maciejewska reported the stability of **oMWCNT#Maciejewska** over three weeks in Dulbecco’s Modified Eagle medium (DMEM) [18]. For covalently obtained gadolinium hybrids with pristine MWCNT, **Gd-L1/MWCNT#Richard**, the minimal concentration of the amphiphilic chelate Gd-L1 was found to be 0.05 mM [16]. The authors reported studies on micelle formation as a mechanism of preventing MWCNT from agglomeration and sedimentation with a critical micelle concentration (cmc) equal to 0.06 mM. On the other hand, in the course of Richard’s studies it was found that Gd-L1 adsorption on the nanotube takes place, thus also this form is adequate for stable dispersion. Apart from visual and indirect judgment of dispersion stability, we performed long-term UV measurements [26]. The characteristic adsorption maximum at 232–233 nm for four oMWCNT sample batches was found to drop by 12–25% but also shifted hypsochromically since the statistics of the functionalization of the nanotubes changed in the dispersions. This method also allowed us to quantify the stability of different hybrids, e.g., the adsorption of **Fe-L3-oMWCNT#Kuźnik** and **Fe-L4-MWCNT#Kuźnik** decreased in the characteristic UV–Vis maximum by factors of 0.0097/h and 0.0147/h, respectively, over the 24 h period of measurement.

### 2 The use of MWCNT hybrids as MRI contrast agents

#### Mechanistic considerations

Magnetic resonance imaging is based on the phenomenon of nuclear magnetic resonance [48]. There are nuclides of non-zero nuclear spins. ^1^H is the most important representative of this group due to its abundance and very strong signals in NMR. For this reason, further consideration will focus on this nuclide. In a high external magnetic field *B*_0_ the nuclear spins orient along field lines. Slightly more than half of the vectors are directed along the field, while the rest are directed in the opposite direction. The distribution is described by Boltzmann’s relations e^−(Δ^*^E/kT^*^)^. Yet, a radiofrequency pulse of a discrete value governed by Larmor’s law (ω = 42.6·*B*_0_) in the NMR spectrometer may excite the parallel spins onto a higher level of anti-orientation. The return to the ground state, called relaxation, could be registered by a receiver as two separate magnetization vector components, i.e., a longitudinal verctor along *B*_0_ described by the relaxation time *T*_1_ and a transverse vector, *T*_2_, perpendicular to *B*_0_. The decay of the NMR signal is observed in two separate processes: *T*_1_ called also spin–lattice relaxation time describes signal intensity disappearance, while *T*_2_, the spin–spin relaxation time is associated with the loss of frequency coherence among spins. MRI incorporates a gradient modification of the static external field *B*_0_ allowing for a three-dimensional image to be recorded. *T*_1_ and *T*_2_ are sensitive to the mobility of water molecules and are specific to certain tissues and liquids in the organism. Since the recording mode of the MRI tomograph could be tuned to the duration of these times (the so-called *T*_1_- and *T*_2_-weighted images), the organs are well visible in the images. However, often lesions are not sufficiently highlighted. For this reason, contrast agents are applied, accelerating spin relaxation and consequently shortening the appropriate relaxation times. The shortened *T*_1_ appears as a brightening on the MRI images, thus *T_1_*-CAs are designated as "positive" while *T*_2_-CAs are "negative" since their action, by acceleration of transverse relaxation, results in the darkening of a particular object. From the diagnostic point of view it is more favorable to obtain a brighter image by using positive CAs, and in this field d- and f-block metal complexes are used depending on their specificity of targeting to desired organs [49–50]. However, the negative CAs, routinely represented by SPIOs [46], also have their targets. The acceleration efficiency is expressed by the relaxivities *r*_1_ and *r*_2_, which are calculated as the reciprocal relaxation time effect caused by a unit millimolar concentration of the CA (Equation 1).

[1]
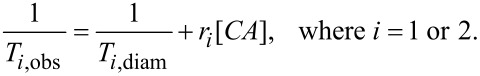


Relaxivity (with a commonly used unit of mM^−1^s^−1^) is a derivative of the magnetic moment of the metal and its complex or the net magnetic moment of the nanoparticle [4]. The Solomon–Bloembergen–Morgan (SBM) theory is a well-established description of relaxation mechanisms, especially for low molecular metal complexes [51–53]. Its formulation is divided into two levels: (i) the inner sphere mechanism, which is most important for the relaxation time *T*_1_ and its *r*_1_ describes the close interaction of water molecules with the paramagnet by direct coordination via neighboring ligands and is given in Equation 2 and Equation 3. These hold for a moderate proton residence time *τ*_M_ ≈ 10 ns with *C* being a constant, *q* the number of inner-sphere water molecules, µ_eff_ the effective magnetic moment, *τ*_C_ the molecular correlation time, *r* the metal–H distance, *τ*_S_ the electronic correlation time, *τ*_M_ the proton residence time, and *τ_R_* the rotational correlation time; and (ii) the outer sphere mechanism, which considers the fact that water molecules diffuse into the density of the resultant magnetic field.

[2]
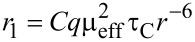


[3]
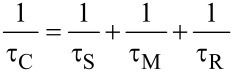


**Figure 4:**
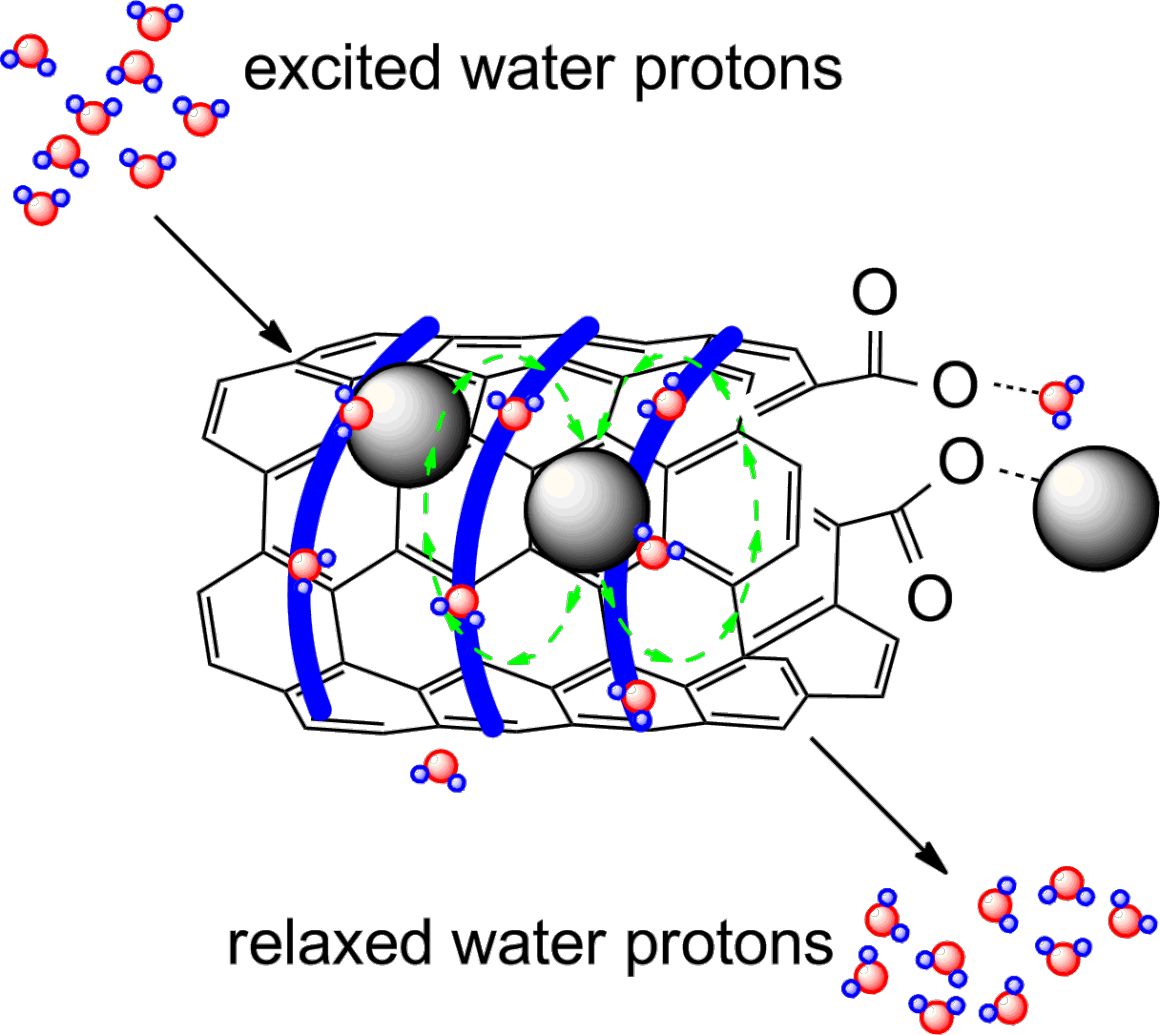
Relaxation enhancement of water protons by the MWCNT hybrid.

The mechanistic discussion on relaxivity measurements for CNT is not developed so thoroughly as is the case for low molecular mass complexes [54]. Several works have relatively easily adapted the SBM theory to relaxation enhancement of single or clustered ions adsorbed on the SWCNT and MWCNT (Figure 4). For example, the SBM theory has been assigned to gadonanotubes (GNT), where gadolinium is supposed to form hydroxide-bridged clusters with many water molecules (*q* in Equation 2) interacting with the paramagnets and consecutively replacing (with *τ*_M_ in Equation 3) one another [3,10]. Generally, a bulky CNT reduces the motion of the paramagnet, thus prolonging *τ*_C_ and therefore increasing the resulting relaxivity [36].

On the other hand, superparamagnetic nanoparticles, both in the most common SPIO and in others (e.g., CoFe_2_O_4_, CdTe quantum dots), anchored on the nanotube net require an extension of the SBM theory [13–14]. Moreover, the hypothesis of CNT as a conducting wire generating its own magnetic field [16,25] with additional contribution of the residual nanoparticles remaining after nanotube synthesis implies the need to create a modified theory at the nano-level. Such a theory that would describe the majority of MWCNT CAs has not been published yet. Nevertheless, there is an experimental and theoretical approach to fill this gap. Koenig managed to derive *T*_1_ and *T*_2_ relaxation time functions for nanoparticles within a broad range of the magnetic field [55]. This theory applies to spherical nanoparticles with a cubic crystal unit, such as SPIO. However, the inhomogeneity (inherent nanocomposite nature) of MWCNT hybrids, their size dispersion and non-uniform shape may cause deviation from the theoretical simulations. Nevertheless, the most important consequences of Koenig’s theory (NMRD simulations) in MWCNT hybrids is their growing *T*_1_ and *T*_2_ acceleration potential oriented upfield, i.e., a bell-shaped curve in a range of ca. 1–400 with a central apogee [56]. It has also been proposed that the relaxivity field dependence effect could be explained by the mobility of MWCNTs, i.e., a high magnetic field keeps them “frozen” and thus less efficient than CAs [18]. Another implication is the enhanced relaxivity of the size of the nanoparticles. These relations are also in general agreement with SBM theory. A more practical hint resulting from the theory is the design of a nanoparticle *T*_1_ CA exhibiting a high *T*_1_ acceleration over *T*_2_ by keeping the ratio *r*_2_/*r*_1_ to about 1–2. This approach could be achieved by synthesizing a uniform size of the nanoparticles [17,56–57].

#### Relaxivity in vitro

First, quantitative validation of the MWCNT hybrids as MRI CA candidates is performed by measuring their relaxivities. According to Equation 1, it is an effect of relaxation time shortening (an increase of the relaxation rate) by the contrast agent. This value is commonly used to compare the candidates at the stage of research as well as to reference new models to clinically approved formulations [58–59]. On the other hand, there are numerous parameters that influence the final relaxivity results. For instance the dependence on the magnitude of the magnetic field could be studied with NMRD measurements. We assume that the measurement techniques, i.e. pulse sequence inversion recovery (IR) for *T*_1_ and Carr–Purcell–Meiboom–Gill (CPMG) for *T*_2_ with their specific parameters as well as the elimination of the radiation damping problem, have all been adapted by spectroscopists and thus have only a marginal effect on the results [60]. Relaxation acceleration is observed along with increasing temperature within the room–body temperature range [61]. However, the temperature decreases the viscosity and increases diffusion and mobility. These effects are partially taken into account in the diamagnetic factor of Equation 1 (*T_i_*_,diam_). However, they also affect relaxivity in a non-linear way. Finally, the medium used for the preparation of dispersion samples and the following dilutions have multiple effects on the relaxivity measurements: in the case of carbon nanoparticles, any additives to water, such as a polymeric substance, will increase the stability of dispersion by reducing agglomeration by wrapping the particles. However, this leads to loading the CNTs with an additional mass and decreasing their mobility. According to the classical SBM theory (τ_R_ in Equation 3), an elevated relaxivity could be observed. On the other hand, the presence of a strong electrolyte in the buffers (PBS, saline) leads to an additional destabilization of the dispersion by the salting effect. These considerations, although important for the relaxivity measurements, can be treated marginally as long as the reference measurements are performed under the same conditions. Since this is a rare case in the presented data, the in vitro relaxivity (see below in Table 1) results should be treated only as an assessment of the potential effect. Nevertheless, the in vivo measurements provide a more pragmatic view of the usefulness of particular models. There, additional effects appear and the relaxivity values are validated by the visual contrast.

Additionally, the way of conducting relaxivity calculations requires comment. Relaxivity is intended to express the acceleration effect of the appropriate relaxation time (*T*_1_ or *T*_2_) caused by interaction with a non-diamagnetic agent. Now, for complexes with low molecular mass molarity is an easy and convenient value to operate with. This way of calculating relaxivity was translated onto the nanostructured bodies. However, only the magnetic centers (Gd, Fe, Co, Mn, and other lanthanides) are in fact the "active sites" for relaxation altering, and this assumption is consistent with expressing relaxivity with the use of molarity. Nevertheless, the total mass of the CA nanostructure, treating CNT as a carrier of the magnet, appears indirectly in the percentage of metal of the body. However, we are convinced that valuable information would be a relaxivity calculated on the total mass of the CA, since the mass will matter in designing the dose and the load to the organism. It is also important when designing the appropriate dispersions since their concentration takes into account mainly the mass of the CNT. For this reason, we calculated such relaxivity results (in (mg/mL)^−1^·s^−1^) and added them to the gathered data set (Table 1). This was possible only for cases in which the percentage of magnet material on the CNT and the dispersion concentrations were both given.

**Table 1:** In vitro relaxivity of MWCNT hybrids.^a^

MWCNT hybrid	*l*/*d* (μm/nm)	% Fe	*r*_1_		*r*_2_		medium	*B*_0_ (T)	*T* (°C)	*C* (μg/mL)	ref.
			(mM·s)^−1^	(mg/mL·s)^−1^	(mM·s)^−1^	(mg/mL·s)^−1^					

**Gd-L1/** **MWCNT** **#Richard**	0.5–2.0/ —	—	12–50	—	—	—	water	0.5	25	1000	[16]
			2–4					11.8			

**CdTe-SPIO-** **oMWCNT** **#Chen**	—	—	—	—	—	—	—	3	—	—	[40]

**MSC/Pol/** **MWCNT** **#Vittorio**	2.0/ 20–40	2.6	—	—	564	260	1% agarose gel	7.1	19	10–180	[22]

**SPIO/** **oMWCNT** **#Wu**	—	30	—	—	176	939	water	3.0	rt	—	[38]

**CoFe_2_O_4 _/oMWCNT #Wu**	0.5–2.0/ 10–30	17	7	21	191	595	water	0.5	—	—	[39]
			—	—	153	475		3.0			

**EuGdLa/** **SiO_2_/** **PAH/** **MWCNT** **#Chen**	—	—	1	—	16	—	water	1.5	—	—	[25]

**oMWCNT** **#Ding**	1.0/ 10–50	1.3	—	—	489	110	1% Pluronic^®^ F127	1.5	—	50–300	[35]
		1.7	—	—	480	147					
		2.1	—	—	401	147					
		2.9	—	—	555	290					

**PEI/PSS/** **oMWCNT** **#Yin**	0.1–0.2/ —	—	—	—	264	—	water	3.0	—	—	[34]

**SPIO-L2-** **oMWCNT** **#Lamanna**	1.5/ 1.0	—	13	—	103	—	water	0.5	37	—	[37]

**SPIO@Lac-Gly/PDDA /oMWCNT #Liu**	10.0/ 40–60	—	—	—	186	—	agarose gel	3.0	—	—	[32]

**SPIO/** **oMWCNT** **#Wang**	—	15	—	—	425	1141	1% agarose gel, 0.5% Pluronic^®^ F127	7.0	—	—	[43]

**oMWCNT** **#Kuźnik**	1.0/ 10–50	2.2	—	—	102	40	1% SDBS solution in water	0.4	37	63–1000	[41]
**Fe-oMWCNT** **#Kuźnik**		13	—	—	18	40					
**oMWCNT** **#Kuźnik**		2.2	0	1	64	25		7.1	22		
**Fe-oMWCNT** **#Kuźnik**		13	0	0	15	35					

**Gd-DTPA-** **oMWCNT** **#Marangon**	0.4/ 20–30	7.1	23	21	13	12	water, agarose gel	0.5	25	—	[36]
			4	3	5	5		4.7			

**Fe-PEG-** **oMWCNT** **#Kuźnik**	1.0/ 10–50	13	—	—	18	41	water	7.1	22	50–500	[26]
**Fe-L3-** **oMWCNT** **#Kuźnik**		16	—	—	22	64					
**Fe-L4-** **MWCNT** **#Kuźnik**		6	—	—	52	58	1% SDBS solution in water				

**oMWCNT** **#Maciejewska**	1.0/29	1.6	—	—	130	36	water	0.4	37	6–300	[18]
	1.0/49	2.3			165	69					
	1.0/40	5.0			121	107					
	1.0/29	1.6			136	38	fetal bovine serum				
	1.0/49	2.3			86	36					
	1.0/40	5.0			110	98					
	0.5/29	1.6			171	48	water				
	0.5/49	2.3			61	25					
	0.5/40	5.0			44	39					
	0.5/29	1.6			22	6	fetal bovine serum				
	0.5/49	2.3			24	10					
	0.5/40	5.0			41	37					

**PM-b-PEG/** **SPIO** **@oMWCNT** **#Liu**	1.0–10.0/ 50–60	—	—	—	85	—	—	3.0	—	—	[32–33]

**Gd[DTPA]^2−^** **clinical** **Gd CA**	—	—	4.1	—	4.6	—	water	1.5	—	25	[32,36]

**Ferrumoxtran-10** **clinical** **SPIO CA**	—	—	9.9	—	65	—	water	1.5	—	—	[17]

**Endorem** **clinical** **SPIO CA**	—	—	—	—	325	—	1% agarose gel, 0.5% Pluronic^®^ F127	7	—	—	[43]

^a^*l*/*d*: length/diameter, % Fe: iron content (m/m); *r*_1_ and *r*_2_: relaxivities (see Equation 1); *B*_0_: magnetic field during measurements, for ^1^H the frequency (in MHz) could be calculated by multiplication of the magnetic field with a factor of 42.5; *T*: temperature; *C*: concentration of MWCNT.

#### *T*_1_ contrast agents

The gadolinium-enriched hybrids **Gd-L1/MWCNT#Richard** [16] and **Gd-DTPA-oMWCNT#Marangon** [36] have the most prominent *r*_1_ results. They were designed for this purpose by chelating Gd^3+^ with the DTPA ligand, which is known as the classical gadolinium CA [62]. Contrary to GNT, where gadolinium cations are chemically adsorbed onto the oxidized scaffold of SWCNT forming oxygen-bridged clusters with very high *r*_1_ (≈150 mM^−1^s^−1^, 1.5 T, 37 °C in Pluronic^®^ F98) [3], here the ligands are responsible for stable coordination of the gadolinium cation. In the case of **Gd-DTPA-oMWCNT#Marangon**, gadolinium ultrastructures were found in the defects of the graphene layer. Their relaxivity is multiple times higher than for the [Gd(DTPA)]^2−^ complex. There could be several reasons for these results. The main factor seems to be the tumbling time of the hybrid, which is much higher than that of [Gd(DTPA)]^2−^. This, according to Equation 2 and Equation 3, is related to the increase of relaxivity. For the oxidized CNT, **Gd-DTPA-oMWCNT#Marangon**, the presence of oxygen functional groups could explain the elevated relaxivity due to hydrophilicity resulting in a more effective water exchange (*τ*_M_ in Equation 3) and the higher number of water molecules in the vicinity. Some structural factors may serve to additionally enhance the relaxivities. In the CNT net, shorter Gd–O bonds were found and this also translates into a stronger Gd–OH_2_ interaction [3,63]. Since relaxivity drops along with the sixth power of the distance to the paramagnets, the closer approach of the water molecule to the paramagnetic center is seriously advantageous. And, finally, which could also be valid for the non-oxidized CNT, **Gd-L1/MWCNT#Richard**, the entropic effect of several neighboring paramagnetic centers interacting simultaneously with single water molecules translates into its faster relaxation [63]. A distinct feature of these hybrids is a high *r*_1_ equal to their *r*_2_. As was mentioned before, an *r*_2_/*r*_1_ ratio in the range of 1–2 is recommended for *T*_1_ CA [4,57]. This is not valid for other hybrids with elevated *r*_1_, such as **CoFe_2_O_4_/oMWCNT#Wu**, **EuGdLa/SiO_2_/PAH/MWCNT#Chen** and **SPIO-L2-oMWCNT#Lamanna**, where the ratio exceeds 8. Based solely on the relaxivity results, they seem to serve better as *T*_2_ CAs, however, in vivo validation is important since there is a report of relaxivity quenching in vivo [36]. A high *r*_1_ candidate once introduced into the cells lost its efficiency in *T*_1_ acceleration related to the iron content but maintained its *r*_2_. Both hybrids, **Gd-L1/MWCNT** and **Gd-DTPA-oMWCNT#Marangon**, express a relaxivity decrease along with a growing magnetic field, but still a higher and less field-dependent one than [Gd(DTPA)]^2−^.

#### *T*_2_ contrast agents

**Figure 5:**
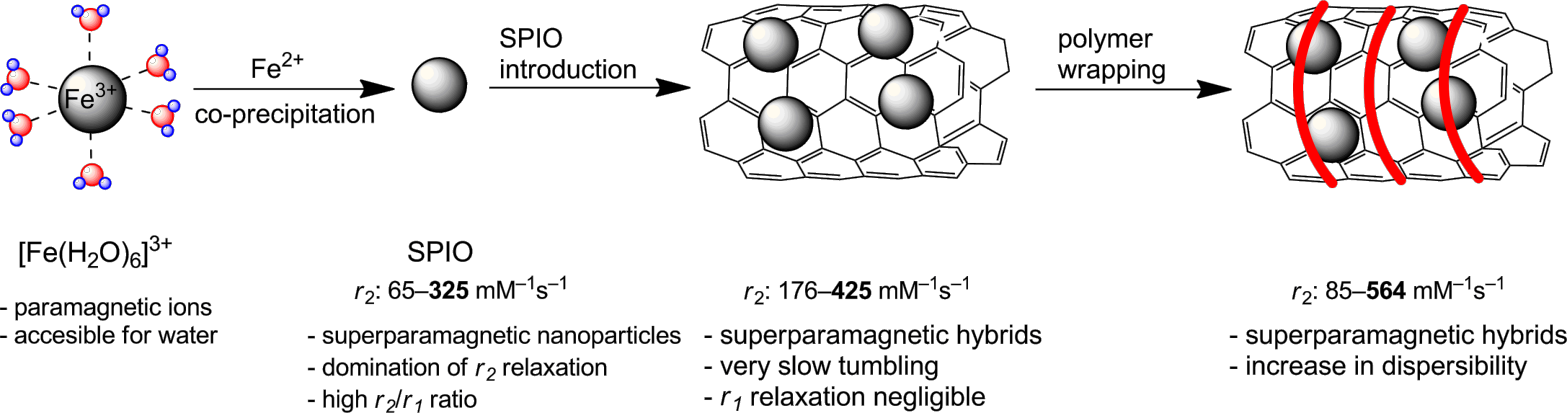
Relaxivity evolution by transformations of iron complexes to SPIOs and forming MWCNT hybrids. The relaxivities in bold face type highlight the trend of an increasing *r*_2_ value.

The evolution of magnetic properties and the resulting *r*_1_ and *r*_2_ of iron and its hybrids are presented in Figure 5. Typical paramagnetic Fe^3+^ ions of *r*_1_ = 0.94, *r*_2_ = 1.06 mM^−1^s^−1^ (water, 0.47 T [64]) become superparamagnetic SPIOs once co-precipitated with Fe^2+^ under basic conditions, increasing *r*_2_ to 65 mM^−1^s^−1^ and more while *r*_1_ = 9.9 mM^−1^s^−1^ [17,65]. Then, anchoring of the SPIOs on the MWCNT results in a further *r*_1_ decrease by hampering local interaction with the water molecules, while the “size effect” leads to higher *r*_2_ [32,37]. The net effect of this last transformation leads to an increase of the *r*_2_/*r*_1_ ratio, which is a desired effect for a *T*_2_ CA MRI candidate. Further coating with polymeric surfactants may decrease *r*_2_ (**PM-b-PEG/SPIO@oMWCNT#Liu**). However, it has other serious advantages, e.g., stability of dispersion in buffers, lower cytotoxicity, visual contrast or selectivity for certain organs [33]. Finally, additional effects appear after introduction into a living organism, thus it is not surprising that further evolution of *r*_2_/*r*_1_ takes place there. It was found that the local environment of cells further enhances the ratio for SPIOs, leading to an even better negative contrast [66]. On the contrary, it is a challenge to design a good, nanomolecular positive contrast agent. Despite the initial reports on the positive trend of CNT purification from residual iron left after the catalyst on relaxivity [17], later studies by Doan on SWCNTs [67] and by others on MWCNTs proved that *r*_2_ is strongly related to the iron content within the nanotube [18,32,35]. Maciejewska provided other interesting results of the nanotube length relation to *r*_2_ [18], e.g., **oMWCNT#Maciejewska**, 0.5 and 1.0 µm in length and with various iron content, was compared. In the majority of cases the longer hybrids had higher *r*_2_. This observation is consistent with the relaxation theory, where a longer, heavier body tends to tumble slowly (Equation 2 and Equation 3) and thus exhibits higher values of *r*_2_.

The highest *r*_2_ results (Table 1) are those registered in the poloxamers (Pluronic^®^), **oMWCNT#Ding** and **SPIO/oMWCNT#Wang**, or in agarose gel (**MSC/Pol/MWCNT#Vittorio**). The relaxivity here is higher than that for Endorem^®^, which is a routinely used SPIO *T*_2_ MRI CA. The non-ionic surfactants gave doubled relaxivity in studies on pristine SWCNTs as compared to ionic surfactants such as SDBS [19]. This effect resulted from better access of the water molecules to the nanotube surface, especially to the end of the CNT, where the residual iron nanoparticles are mainly located. The helically wrapping non-ionic surfactants seem to enable such water interactions. On the contrary, ionic surfactants tend to form micelles that limit access for the water molecules. Therefore, the results of the SWCNT studies give a general idea of the impact of surfactants on the relaxivity results. When comparing solely *r*_2_ measured in vitro, it is difficult to derive any more general theory of the relation of MWCNT functionalization to relaxivity. Moreover, unstandardized measurements, which are sensitive especially to the strength of the magnetic field, make it even less clear. Since the general trend is a relaxivity decrease in higher magnetic fields, it seems to be valuable to highlight the highest *r*_2_ in fields above 1.5 T [18,41]. This is additionally supported by the trend of introducing high-field scanners into clinical use. Thus models exhibiting high relaxivity in fields of 3.0 T and above should be especially attractive. Therefore, **CoFe_2_O_4_/oMWCNT#Wu**, **SPIO/oMWCNT#Wu** and **PEI/PSS/oMWCNT#Yin** constitute the most promising group of hybrids. Decoration of the oxidized MWCNT with superparamagnets or with a set of polyelectrolytes, with the most negatively charged layer on the final, outer surface, resulted in the best efficiency of relaxation enhancement. **SPIO/oMWCNT#Wu** is also an interesting hybrid due to the highest *r*_2_ in water calculated on the total mass of the hybrid, i.e. (mg/mL)^−1^s^−1^. This result translates into a lower dose of the potential nanostructured CA being administered to the patient.

### 3 In vivo effects

An assessment of the in vivo behavior of nanohybrids is essential in order to judge their applicability in MRI. Several types of studies were performed. The first group was to study cytotoxicity by determining cell viability upon incubation with the nanohybrids (Table 2). In some cases it was possible to indicate which component of the hybrid was responsible mainly for the cytotoxicity impact. The classical MTT assay is usually applied for this purpose. However, there have been reports of high measurement error in this method with MWCNT [40], thus Trypan Blue staining of the dead cells could be more reliable. Additionally, the Alamar Blue test was also used [32,37]. For administration by vein injection, hemolysis studies are also useful and give a general idea as to blood compatibility. Then, biodistribution and elimination sketch further the directions for the applications. An analysis of organs with an MRI scanner in vivo (see below in Table 3), TEM and ICP post mortem together with studies of the urine and fecal samples give a broader view and verification of the safe and effective action of the CA candidate. Final observation of animal behavior after administration, although performed only for a short period, is consistent with the abovementioned toxicity studies.

**Table 2:** Cytotoxicity of MWCNT hybrids.^a^

MWCNT type	model	cell culture	viability (%)	time (h)	concentration (µg/mL)	ref.

**CdTe-SPIO-oMWCNT#Chen**	hybrid	HEK 293T	99, 30	24	2, 15	[40]
	reference		99, 97			
**SPIO/oMWCNT#Wu**	hybrid	MCF-7, L929	80	24–48	200	[38]
**CoFe_2_O_4_/oMWCNT#Wu**	hybrid	HeLa	98, 80	24	25, 200	[39]
		L929	92, 84			
**SPIO-L2-oMWCNT#Lamanna**	hybrid	PC3	100	20	2–10	[37]
**SPIO@Lac-Gly/PDDA/oMWCNT#Liu**	reference	HEK 293	58	72	150	[32]
		Huh 7 Cell	100			
	hybrid	HEK 293	100			
		Huh 7	99			
**oMWCNT#Maciejewska**	3.9% Fe	fibroblast	84, 50, 41	24	5, 100, 600	[18]
	5.8% Fe		98, 67, 1			
	12.4% Fe		82, 51, 46			
	3.9% Fe	HeLa	100, 97, 73			
	5.8% Fe		97, 96, 59			
	12.4% Fe		100, 86, 53			
**PM-b-PEG/SPIO@oMWCNT#Liu**	hybrid	CHO-GFP	94	72	200	[33]
		Huh 7	91			

^a^Model: results are given for a particulate MWCNT hybrid, in some cases results for a reference oMWCNT were provided, different iron content in **oMWCTN#Maciejewska** hybrids was investigated; viability: multiple values refer to different concentrations.

#### Cytotoxicity and hemolysis

Cytotoxicity of the nanohybrids was studied on various cell types (HeLa, HEK 293, human prostate cancer cells PC3, fibroblasts and others) and a general conclusion is the dose-dependent trend. However, the relations are more complicated (Table 2). Maciejewska investigated oMWCNTs with different iron content (3.9, 5.8 and 12.4% Fe (m/m)) and found that HeLa cells were more viable upon treatment with the iron-poorest oMWCNT, while fibroblasts expressed the highest viability in the case of nanotubes with medium iron content [18]. On the other hand, mitochondrial staining allowed us to analyze cell pathogenesis. There, again the iron-poorest oMWCNTs left the organelle intact, while others led to shape alteration, from typical tubular to globular forms. A higher concentration, above 300 µg/mL, led to a further deviation of the iron content–viability trends. Liu assigned the viability loss to agglomeration of the nanotubes [32]. Thus further functionalization with polymers is favored and a promising viability could be achieved even with high concentrations of **PM-b-PEG/SPIO@oMWCNT#Liu** reaching 200 µg/mL [33]. For **CdTe-SPIO-oMWCNT#Chen**, the main viability loss was found to come from cadmium [40]. In this regard, an important observation was made by Lamanna, i.e., that covalent bonding of nanoparticles on the oMWCNT is permanent even upon processing by the cells [37]. **SPIO/oMWCNT#Wu** and **CoFe_2_O_4_/oMWCNT#Wu**, within concentrations up to 400 µg/mL, did not express hemolytic activity higher than 5% [38–39].

Several toxicity studies of the hybrids in animals have already been reported. **SPIO@Lac-Gly/PDDA/oMWCNT#Liu** was injected into mice (tail vein) at a dose of 10 mg/kg. Short-term discoloration of the tails was observed, but no other remarkable changes were recorded over the next five days [32]. Thus, no acute toxicity was associated with the hybrids. A polymer-functionalized hybrid, **PM-b-PEG/SPIO@oMWCNT#Liu**, was applied with a 10-fold dose in similar studies with the same effect [33]. Five mice expressed 100% survival without any remarkable side effects after intramuscular injection of **Gd-L1/MWCNT#Richard** at a dose of 0.1 mM Gd/kg [16].

#### Biodistribution

The desired effects of biodistribution are observed in MRI scanners. These are described in the section “Magnetic resonance imaging”. However, here a brief report on the post mortem analysis of animal organs is presented in order to give an idea of the clearance routes and periods of the nanohybrids. **SPIO/oMWCNT#Wu** was injected into the tail veins of KM mice. After administration, the nanohybrid was taken in mainly by the heart, liver, spleen and lung, maintaining a constant level (±20%) for 8 h, however, no obvious damage to the organs was noticed [38]. CNT deposits were observed in the spleen and liver, but there were also some minor traces in the kidneys. CNTs with iron were found in the feces, but only iron was detected in the urine. These observations confirmed that the majority of hybrids had been eliminated via the feces. On the contrary, **Gd-DTPA-oMWCNT#Marangon** was found to undergo renal clearance and no lung accumulation was observed [36]. The presence of MWCNT CA candidates has not yet been tested in the brain. However, they are known for their ability to penetrate the blood-brain barrier, particularly as a function of their diameter [68].

#### Magnetic resonance imaging

The visual effect of the MRI CA candidate constitutes final verification which is most important for this technique. The quantitative results are provided in Table 3.

**Table 3:** In vivo MRI contrast effect of the MWCNT hybrids.^a^

complex	*B* [T]	place	SI [%]	time	dose	ref.

**MSC/Pol/MWCNT#Vittorio**	7.1	MSC	77 (*T*_2_)	5 days	1 µg/mL	[22]
		MSC	106 (*T*_1_)		1 µg/mL	
**SPIO/oMWCNT#Wu**	3	MCF-7 cells	9	3 h	100 µg/mL	[38]
			68		10 µg/mL	
			100		0 µg/mL	
		liver, mice	37	15–300 min	2.5 mg/kg body	
		spleen, mice	75			
**CoFe_2_O_4_/oMWCNT#Wu**	3	HeLa cells	15	3 h	100 µg/mL	[39]
			58		10 µg/mL	
			100		0 µg/mL	
**oMWCNT#Ding**	1.5	tumor in mice grown from MDA-MB231 cells	28	10 min	100 µg per tumor	[35]
			19	1 day		
			15	7 days		
**PEI/PSS/oMWCNT#Yin**	3	HeLa cells	55	6 h	0.08 mM Fe	[34]
			36			
**SPIO@Lac-Gly/PDDA/oMWCNT#Liu**	3	intravenous injection, liver, mice	31	right after injection	10 mg/kg body	[32]
		intravenous injection, tumor, mice	117			
**PM-b-PEG/SPIO@oMWCNT#Liu**	3	intravenous injection, liver, mice	61	right after injection	100 mg/kg body	[33]
		intravenous injection, tumor, mice	93			

^a^SI: Signal intensity enhancement after injection.

The MWCNT hybrids were first analyzed in an MRI scanner in a simple in vitro experiment in which various concentrations of the hybrids were arranged in a row. A visual change in color intensity according to the concentration gave a positive impression [25]. The hybrids turned out to be most efficient as contrast agents in the *T*_2_-weighted technique. There, darkening of the image was observed. A similar approach was taken to assess the contrast effect on the cells, where darkening of the image caused by the MWCNT hybrids was recorded in reference to untreated cells. Cancer cells exhibited from 9 up to over 100% signal intensity enhancement caused by the hybrids. For medical diagnosis, 10% signal enhancement is already sufficient to observe a visually significant contrast. **PEI/PSS/oMWCNT#Yin** hybrids were tested on HeLa cells and compared to hybrids functionalized additionally with FA [34]. Since HeLa cells exhibit high over-expression of the folate receptor, the targeting effect was remarkable and was found to reach 64% signal enhancement in reference to 45% for the non-functionalized hybrids. Lamanna and Vittorio were able to localize the marked cells by their darkened contrast and emphasized the unique features of MWCNTs able to penetrate the cell membrane with future applications as theranostic systems (**thera**py + diag**nosis**) [22,37].

Finally, studies on mice allowed for real in vivo verification of both the contrast potential and biodistribution as related to affinity to specific cells and clearance routes. Signal intensity enhancement is calculated as the ratio of intensity growth before and after injection. Intramuscular injection into one of the legs of the mice gave a negative contrast, even for the gadolinium **Gd-L1/MWCNT#Richard** hybrid [16], while gadonanotubes are known to be very efficient positive contrast agents [14]. In Liu’s models, selectivity to tumors is clearly visible by specific contrast enhancement [32–33]. For comparison, Magnevist^®^, a classic positive contrast agent, led to similar contrast of both the liver and the tumor. Additional features of oMWCNTs were studied by Ding, as the nanotubes were exploited in laser-induced thermotherapy (LITT) in mice [35]. Because MWCNTs are better energy absorbers than other CNTs, they turned out to be efficient transducers delivering an appropriate thermal dose to the tumor target area with a parallel imaging modality. Such polymodal systems constitute one of the trends for further CA development.

## Conclusion

### The recipe for a promising MWCNT MRI contrast agent

As a conclusion we would like to propose a model of an MWCNT hybrid as a promising MRI CA candidate. The proposed design is based on the results presented in this review. We would like to emphasize that there is no single universal solution for such a model due to different targeting and various other expectations, such as efficiency in other modalities, such as computer tomography or chemiluminescence. Thus our intention is to sketch out the possible directions of the transformations and functionalizations of MWCNTs. Based on Maciejewska’s and Ding’s studies, the most iron-rich MWCNTs should be obtained at the stage of production. Size is another parameter that should be adjusted to the expected targeting (penetration of the cell membrane or more hydrophilic systems for angiography). Since oxidation of pristine MWCNTs opens up many possibilities for further (non-)covalent functionalization, this is most likely the best route for MWCNT pretreatment so far [69], however, covalent functionalization could also lead to good results. Then functionalization with a polymer is necessary, while the way of decoration (covalent or non-covalent) seems to be less important, as the character of the surfactants plays a more important role. Non-ionic poloxamers (e.g., Pluronic^®^) or their "custom-made" counterparts have already given the best results, both in contrast effect and in low cytotoxicity. Finally, decoration of superparamagnetic nanoparticles seriously enhances the relaxation effects. It is too early to judge whether covalent bonding is also necessary, but what is important is the uniform nature (size, structure, composition) of these nanoparticles. The last feature is responsible for selective function in MRI (positive or negative contrast). GNT and our models show a promising approach for CNT decoration with single paramagnetic ions or their clusters to obtain well spread and therefore efficient relaxation enhancers. In parallel, other functionalities could be considered, designed and carefully introduced onto the nanotube scaffold.

### Future MWCNTs as MRI CAs

There are several development routes for MWCNTs as MRI CAs. One of them is endowing hybrids with an additional modality. Such examples already belong to the models that were described in this review. However, new combinations have been reported very recently [70] and the multiplication of modalities in one agent is a challenge, with "six modalities-in-one" being the winner so far [71]. Yet, molecular probes penetrating a living organism and emitting an appropriate signal in case of pathogenesis seem to be within our reach. Another trend seems to be focused on polishing the properties of existing nanomaterials in order to adjust them to the targets, to make them more biocompatible and to reduce any side effects. This is achieved by compositional tuning, size control and morphology design [9]. There the organic ligands, their structure and role are still to be enhanced on the basis of classical interactions and nanostructure impact. At the current stage, many more experiments have to be performed to unify the data, to assess both the acute and long-term side effects and to derive such features of CNTs so as to apply them safely in living organisms. Finally, combining the diagnostic features with therapeutic activity, i.e., creating so-called theranostic systems, opens up new territories for medicine in the 21st century. Such models have already appeared among the MWCNT hybrids, e.g., those coupled with stem cells, those having thermal ablation functionality and those carrying technetium radionuclide. This rapidly growing field of drug delivery will surely exploit MRI as a tracking technique, but also the intrinsic magnetic properties of MRI CAs provide huge possibilities for "magnetically driven" medicine. We hope that these “nano-devices” can, at least in this way, enhance life quality.

## List of Abbreviations

Table 4 gives a list of abbreviations used in the manuscript.

**Table 4:** List of abbreviations.

abbreviation	

acac	acetylacetonate, 2,4-pentanodionate
CA	contrast agent
CNT	carbon nanotube
DTPA	diethylene triamine pentaacetic acid
FA	folic acid
Gly	glycine
GNT	gadonanotubes
Lac	lactose
LBL	layer by layer
MSC	mesenchymal stem cells
MWCNT	multiwalled carbon nanotube
MRI	magnetic resonance imaging
NMR	nuclear magnetic resonance
PAH	poly(allylamine hydrochloride)
PDDA	poly(diallyldimethylammonium chloride)
PEI	polyethylenimine
PEG	poly(ethylene glycol)
PMETAC-*b*-PEGMA	poly[2-(methacryloyloxy)ethyltrimethylammonium chloride]-*block-*poly(ethylene glycol) monomethacrylate
Pol	poloxamer, nonionic triblock copolymers, e.g. Pluronic^®^
PSS	polystyrene sulfonate sodium salt
SDBS	sodium dodecylbenzenesulfonate
SPIO	superparamagnetic iron oxide
SWCNT	single-walled carbon nanotube

## Supporting Information

File 1 The data processed from the cited works.
